# Basic concept and clinical applications of quantitative ultrasound (QUS) technologies

**DOI:** 10.1007/s10396-021-01139-6

**Published:** 2021-10-20

**Authors:** Tadashi Yamaguchi

**Affiliations:** Center for Frontier Medical Engineering, Chiba University, 1-33 Yayoicho, Inage, Chiba 2638522 Japan

**Keywords:** Quantitative ultrasound, Attenuation, Speed of sound, Amplitude envelope, Backscatter coefficient

## Abstract

In the field of clinical ultrasound, the full digitalization of diagnostic equipment in the 2000s enabled the technological development of quantitative ultrasound (QUS), followed by multiple diagnostic technologies that have been put into practical use in recent years. In QUS, tissue characteristics are quantified and parameters are calculated by analyzing the radiofrequency (RF) echo signals returning to the transducer. However, the physical properties (and pathological level structure) of the biological tissues responsible for the imaging features and QUS parameters have not been sufficiently verified as there are various conditions for observing living tissue with ultrasound and inevitable discrepancies between theoretical and actual measurements. A major issue of QUS in clinical application is that the evaluation results depend on the acquisition conditions of the RF echo signal as the source of the image information, and also vary according to the model of the diagnostic device. In this paper, typical examples of QUS techniques for evaluating attenuation, speed of sound, amplitude envelope characteristics, and backscatter coefficient in living tissues are introduced. Exemplary basic research and clinical applications related to these technologies, and initiatives currently being undertaken to establish the QUS method as a true tissue characterization technology, are also discussed.

## Introduction

Ultrasound has been widely used in medicine since its biological effect was confirmed by Langevin in 1917. Ultrasonic diagnostic equipment became widely available in the 1950s and is used in mainly qualitative diagnosis by the visual observation of signal waveforms in A-mode, tomographic images in B-mode, and dynamic images in M-mode. During the period when quantitative evaluation of blood flow became possible using the pulse Doppler method in the late 1960s, further quantitative diagnostic technologies that employed the physical characteristics of ultrasound waves as an index were proposed for development. The full digitalization of diagnostic equipment in the 2000s enabled the development of quantitative ultrasound (QUS) techniques, some of which have been put into practical use in recent years. In QUS, the radiofrequency (RF) echo signals returning to the transducer are analyzed, and parameters that can be used to quantify the tissue characteristics are calculated. In-phase/quadrature-phase (IQ) data are also used instead of RF data.

In this paper, typical examples of QUS techniques for evaluating attenuation, speed of sound, amplitude envelope characteristics, and backscatter coefficient in living tissues are described, and examples are provided of exemplary basic research and clinical applications related to these technologies. Current work under way to establish QUS methods as a true tissue characterization technology are also discussed.

## Attenuation coefficient evaluation

### Overview

Attenuation, defined as the loss of ultrasound signal energy with the propagation depth as a function of frequency, is essential in quantifying tissue properties. Ultrasonic waves that are emitted from the probe and pass through living tissue are reflected and scattered by scatterers smaller than the ultrasonic pulse, pass through mutual interference, and are received by the probe as RF echo signals. The attenuation characteristics of the target tissue can be evaluated by evaluating the backscattered signal that has returned in the same direction as the transmission. The accuracy of the evaluation is dependent on the state of scatterers in the living tissues (e.g., randomness, periodicity, relationship with pulse length), as well as the shape and resolution of the transmitted and received ultrasonic beams.

The attenuation evaluation techniques proposed by numerous researchers over the past decades can be broadly divided into the time domain approach and the frequency domain approach. However, because time and frequency are closely related in echo signals, some techniques use them in tandem. Although the time domain approach is simple and easy to implement, it is inferior in robustness, and accordingly, the frequency domain approach is often used in reality. The attenuation coefficient is the main index used for quantifying attenuation. It should be noted that the unit of the attenuation coefficient in the medical ultrasound field is generally dB/cm/MHz, but dB/m is sometimes used.

### Spectral difference method

The spectral difference method is the most basic technique for evaluating the reduction in the echo signal power spectra along the propagation path of the ultrasound beam, and it has a long development history. Kuk proposed the basic theory of attenuation measurement using broadband pulses in 1978 [1], reported its application to the liver in 1979 [2], and compared it with the spectral shift method in 1985 [3], which is described below. These methods assume that the scattering characteristics to be evaluated are constant and do not change over the depth range. The attenuation coefficient is calculated from the difference or ratio of the power spectra of the signal acquired in advance as reference information (e.g., from a tissue-mimicking phantom) and the signal from regions of interest (ROIs) set at two different depths in the evaluation target. Because of the processing involved, the spectral difference method is sometimes referred to as the reference phantom method. A basic study on the depth dependence of attenuation evaluation using a reference phantom was reported by Yao [4].

### Spectral shift method

The spectral shift method is the most common technique for evaluating the downshift of the echo signal power spectra of an ultrasound beam propagating through living tissues. This algorithm uses the downshift in center frequency of the power spectrum versus propagation depth to estimate the attenuation slope. Local attenuation is evaluated from the attenuation slope of the echo signal in the ROI being evaluated. Regarding the basic methods for determining the attenuation coefficient of tissues, the tissue is generally assumed to have linear frequency-dependent attenuation. In reality, however, many tissues exhibit non-linear frequency-dependent attenuation. Ophir investigated the relationship between downshift of the center frequency of the spectrum and the attenuation coefficient (mechanism of evaluation error) when it is assumed that living tissue with non-linear characteristics is linear, and proposed an in vivo measurement technique with narrow band pulses [5, 6]. Kim reported that the spectral shift between the power spectra obtained at the two different depths was linearly proportional to the product of the attenuation coefficient and to the difference of the depths at which the spectra were obtained, and that this shift provided a direct estimate of the attenuation coefficient [7]. Baldeweck proposed various methods using an autoregressive model for spectral analysis [8], and Fink proposed a method using short-time Fourier analysis [9].

### Hybrid method

In general, spectral shift methods are more robust than the spectral difference methods at the boundary region of backscatter changes; however, they each have specific limitations. Classical spectral shift approaches for estimating ultrasonic attenuation are more sensitive to local spectral noise artifacts and have difficulty in compensating for diffraction effects due to beam focusing. In contrast, spectral difference approaches fail to accurately estimate attenuation coefficient values at tissue boundaries that also possess backscatter variations [10]. Kim proposed a hybrid attenuation estimation method that combines the advantages of the spectral difference and spectral shift methods to overcome the specific limitations of each. The proposed hybrid method initially uses the spectral difference approach to reduce the impact of system-dependent parameters, including diffraction effects. The normalized power spectrum that includes variations caused by backscatter changes is then filtered using a Gaussian filter centered at the transmit center frequency of the system. A spectral shift method employing a spectral cross-correlation algorithm [6] is then used to compute spectral shifts from these filtered power spectra to estimate the attenuation coefficient [10].

### Clinical applications

The high functionality of modern ultrasonic diagnostic equipment has led to the practical application of high-frequency and high-resolution attenuation evaluation methods. Dedicated diagnostic ultrasonic units have been produced for each of the spectral difference, spectral shift, and hybrid methods. The following four types of applications are in current use.

- Evaluation of attenuation based on the frequency shift of the received signal.
- Evaluation of attenuation by comparison with a phantom in which the attenuation and scattering coefficients are known.
- Evaluation of attenuation by comparison with training data obtained with known transmitting and receiving conditions.
- Evaluation of attenuation based on the slope of the ratio of signals transmitted and received at two different frequencies.

Of course, even if the same theory is used, differences exist such as filtering and the combination of several methods depending on the manufacturer and diagnostic equipment. There has been particular interest in the application of attenuation evaluation methods to the field of gastroenterology, in which the results of numerous studies have been reported in the past few years [11–19]. Attenuation evaluation is a classic example of an old technology that has been newly implemented in medical ultrasound. As several methods are used at the same time, the user needs to fully understand the basic theory and limits of the technique used and the meaning of the values presented.

## Speed of sound evaluation

### Overview

When ultrasound waves propagate through the living tissue under observation, the speed of sound is an important parameter that indicates the acoustic characteristics peculiar to a particular living tissue, and it greatly affects the imaging of echo signals in B-mode or M-mode. In general, ultrasonic diagnostic equipment assumes that living tissue is a uniform medium, and it sets the reference speed of sound at 37 °C as 1530 m/s according to the Japanese Industrial Standard (JIS) [1540 m/s according to the American Institute of Ultrasound in Medicine (AIUM)]. However, deviation of the propagation path due to the complexity of the actual tissue structure in living tissue and refraction are not taken into consideration. Therefore, distance measurements based on the received echo signals and the imaged tomographic image will contain errors and distortions. These are considered to lie within the acceptable error range in practical use. However, as the frequencies of ultrasound waves used in diagnosis (and applied to QUS) are continuing to increase, it is necessary to estimate the speed of sound in the local region with greater accuracy. Accurate evaluation of the local speed of sound is also beneficial in calculation of the attenuation coefficient. The following section outlines the techniques for improving image quality by evaluating the speed of sound from the RF echo signal and using differences in the speed of sound at each local region.

### Focusing method

In conventional ultrasound diagnostic equipment, an assumed average speed of sound is used for delay and sum beamforming to create a B-mode image that is generated by the RF signal of each received echo line. Conversely, as proposed by Ogawa and Umemura [20] and Hayashi et al. [21], it is also possible to evaluate the average speed of sound from image quality. In the focusing method, beam focusing is repeated to optimize the local image quality and evaluate the speed of sound at that time. The evaluation indexes of image quality include amplitude as used by Cho et al. [22], minimum entropy as used by Mesdag et al. [23], and lateral sharpness as used by Napolitano et al. and Boozari et al. [24, 25]. Focusing methods are relatively easy to implement, because they directly use the existing hardware configuration of commonly available diagnostic equipment. However, there are restrictions on the conditions for setting the propagation route between the evaluation target and each element of the probe, and the estimation accuracy of the local speed of sound can be low. Methods for solving this problem include the technologies proposed by Jakovljevic et al. [26] and Abe and Kanai et al. [27].

### Spatial coherence method

In the spatial coherence method, spatial coherence is calculated under various conditions, taking into account the number of elements in the probe used for receiving the echo signals, and the speed of sound in a local or wide area is evaluated [28–30]. Since elemental technologies were initially proposed until recently, various phase aberration correction technologies have been proposed for calculating spatial coherence [31–37]. Because this method requires individual control and signal processing for each element of the probe, it has more hardware restrictions compared with focusing methods, which may limit implementation in clinically available equipment. It has the advantages that the area in which the speed of sound is estimated can be set locally or globally, and guaranteed high estimation accuracy of the speed of sound. In addition, Hasegawa has proposed an effective technique that is applicable to both conventional focused imaging using line-by-line transmission/reception and plane wave imaging [38].

### Compounding method

In the compounding method, the speed of sound is evaluated directly by searching for spatial shifts in images between different transmission and reception angles. Specifically, the discrepancy between the speed of sound used for beamforming and the speed of sound of actual living tissue is used as a reference. If the speed of sound mismatch is high, a spatial error occurs, because the optical path length changes when different angles are used. When the magnitude and direction of the shift are optimized, the difference between the assumed speed of sound and the actual speed of sound is minimized [39–43]. The advantage of this method is the direct estimation of the speed of sound; however, it has the disadvantage of low robustness.

### Clinical applications

**Fig. 1:**
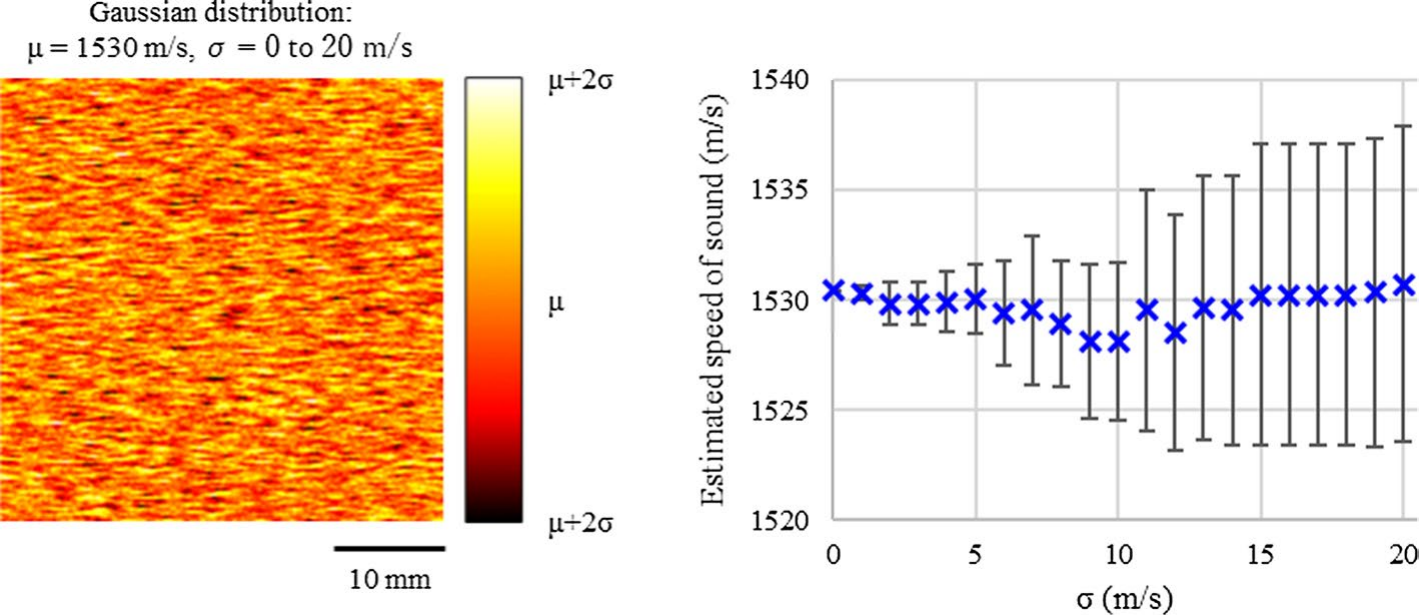
Speed of sound evaluation in a medium that contains multiple types of scattering sources having different speeds of sound. Numerical liver phantom (left) in which the speed of sound of the scattering source varies by an average *μ* and standard deviation *σ*, and results of evaluation (right). This figure was newly created by *N*. Nitta from data reported in Reference [49]

Technologies for evaluation of the speed of sound (and thus image quality improvement) have also been implemented in clinical ultrasound diagnostic equipment, and various applications have been reported. Hirooka has reported the clinical applications of this technology, since the early stages of its development [44]. Imbault has reported clinical data and proposed various methods for improving the accuracy of speed of sound evaluation technology [45–47]. As described by López-Haro [48], this technology is also being used in therapeutic applications. At present, compounding methods have not been implemented in clinical equipment. The high accuracy of signal processing in current diagnostic equipment has benefitted methods used for evaluating the speed of sound. Further developments expected in the future include improvements in accuracy in evaluating the speed of sound and image quality by utilizing acoustic physical quantities. In one of the most recent studies in this area of research, Nitta used a computer simulation to verify the accuracy of speed of sound evaluation for a medium such as the liver, which contains multiple types of scattering sources that have different speeds of sound, as shown in Fig. 1 [49].

## Amplitude envelope statistics

### Overview

In actual living tissues, signals from a small target tissue can become buried by scattered signals from the minute scatterers that are often randomly and densely present over a wide area of surrounding tissues in the medium. In a situation where there are 10 or more scatterers in the resolution cell, which is the resolution of the ultrasound beam, an extremely weak scattering signal is generated in each microscatterer, and received by the probe. The received signal includes a noise signal produced as the result of their interference. Accordingly, a speckle pattern is observed in the final B-mode image. The size of the mottled speckle pattern is determined by the sound field characteristics of the irradiated ultrasound, and there is no correlation between the structure of scatterers in the living tissue and the speckle pattern. In other words, if the observation area contains speckle, then the tissue is in a dense and homogeneous state. Many researchers have proposed the use of probability density functions to express the properties of RF echo signals that exhibit a speckle pattern. Amplitude envelope statistics is a QUS method that normalizes the amplitude envelope characteristics of RF (or IQ) echo signals with the probability density functions. This technology has a long history with ultrasonics in medicine.

### Rayleigh distribution

The Rayleigh distribution, which is a probability density function [50], has been applied to the field of ultrasound as a mathematical model for showing the amplitude envelope characteristics of echo signals that exhibit speckle. This basic model has been widely applied both in basic studies and in clinical applications following its verification with clinical data by Burckhardt [51], and evaluation of application conditions by Wagner [52]. Focusing on the fact that the deviation from the Rayleigh distribution in living tissue indicates inhomogeneity of the tissue, several attempts have been made to realize QUS by indexing the degree of non-Rayleigh distribution. Representative examples of this work include techniques that use signal-to-noise ratio (SNR) as the index, as proposed by Shankar [53, 54] and Fujii [55]; and methods that use variance as the index, as proposed by Kamiyama, which have been implemented in clinical devices [56]. Yamaguchi and Hachiya have proposed multi-Rayleigh distributions that combine two or three Rayleigh distributions to eliminate the constraint of expressing the amplitude envelope characteristics of the echo signal only by the Rayleigh distribution [57, 58].

### Higher order distributions

The Rice distribution, which was proposed by Nakagami [59] and by Rice [60] as a model of wave propagation, describes the diffuse signal component due to a high density of random scatterers. In 1986, Insana proposed its use in combination with a coherent signal component [61]. K-distribution was first introduced by Lord [62] in the context of random walks. K-distribution corresponds to a variable density of random scatterers, with no coherent signal component, and was introduced to ultrasound imaging by Shankar [53, 63], and by Narayanan [64]. Homodyned K-distribution was introduced by Jakeman [65] to model weak scattering. In 1994, Dutt and Greenleaf verified that homodyned K-distribution corresponds to the general case of a variable effective density of random scatterers with or without a coherent signal component [66]. The Nakagami distribution, defined by Nakagami [67, 68], is highly versatile, being applicable in cases where the scatterer to be evaluated is sparse or dense, and also in cases where scatterer density has periodicity. The series of studies conducted by Shankar [53, 63, 64, 69, 70] is useful for comparing these statistical models.

### Clinical applications

The QUS technique based on the Rayleigh distribution has already been implemented in clinical equipment, primarily for the purpose of assessing liver fibrosis [56, 71–73], and it has also been applied to other diseases [74]. In subsequent development, Kuroda proposed the effectiveness of this technique for evaluating steatosis [75], which was verified pathologically and using MR microscopy by Lee [76].

K-distribution and homodyned K-distribution are of particular value in evaluating tumors. In a pioneering study, Shankar developed a method of breast tumor classification for clinical application [53, 54] using K-distribution, and Hao applied homodyned K-distribution for the characterization of cardiac tissue [77]. Mamou and Oelze developed a technique for evaluating tumors in lymph nodes in three dimensions [78, 79]. Omura and Yamaguchi developed a tissue characterization method for the follow-up of healing in ulcers by diagnosing the properties of collagen fibers [80], which is evolving as a technique for evaluating the histological properties of skin diseases, including lymphedema.

**Fig. 2:**
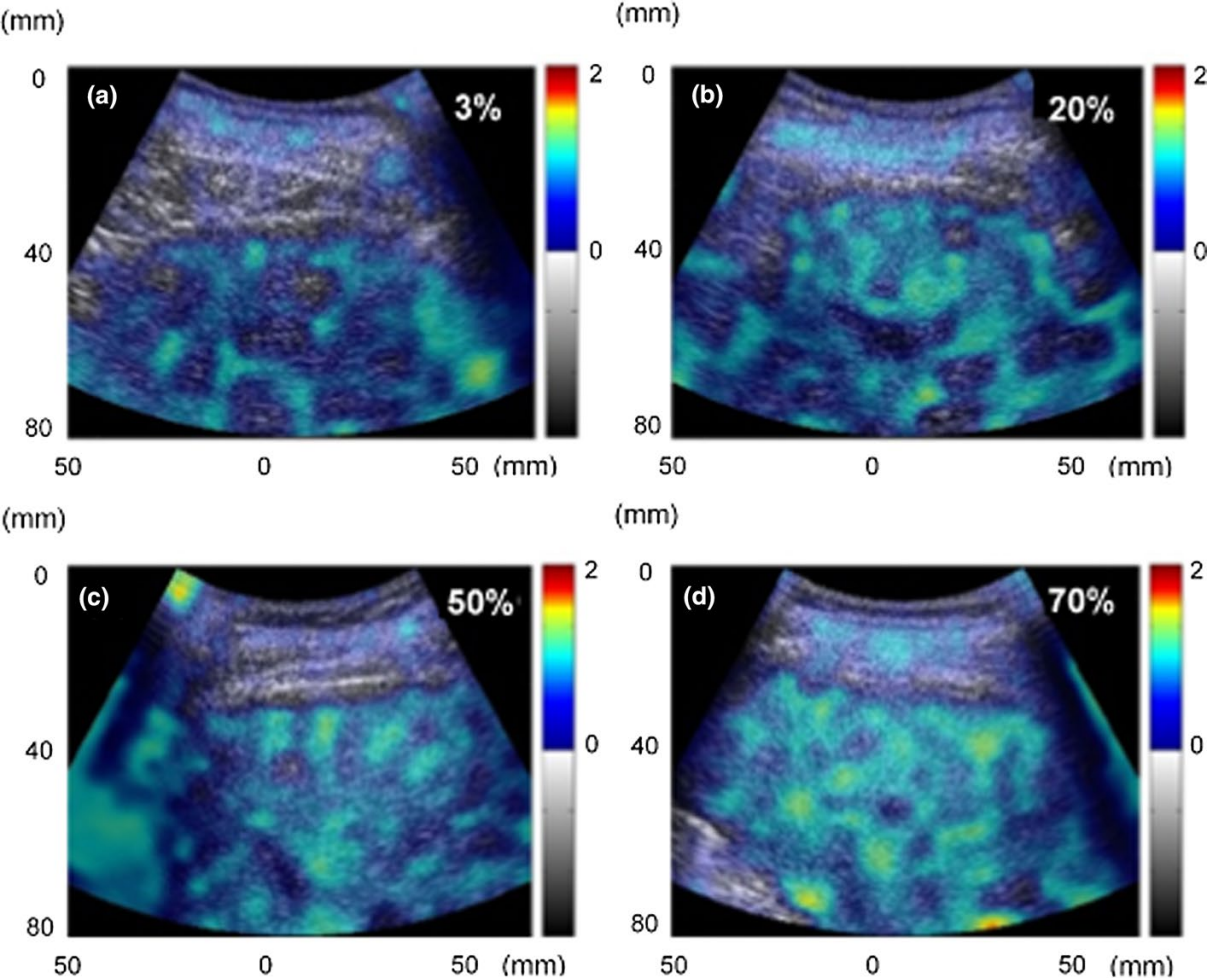
Parametric images of the double-Nakagami distribution parameter for various grades of hepatic steatosis. The color bar indicates the density of adipose tissue

Currently, basic studies that use clinical data most commonly employ the Nakagami model. A wide variety of targets have been evaluated in such studies, including vascular studies by Huang [81], ophthalmology and breast cancer by Tsui [82, 83], and the liver by Tsui and Yamaguchi [84, 85]. In the most recent research, the Nakagami distribution has been applied to evaluating the temperature of living tissues by Hasegawa [86, 87], and Tamura and Yamaguchi have combined multiple distributions to evaluate fat and fiber in the liver simultaneously [88, 89]. Figure 2 shows an example of the evaluation results of liver steatosis using the double-Nakagami model, a complex probability density function that enables quantification of the degree and distribution of fat mass in the liver [84]. The images in the figure indicate that the amount and density of adipose tissue (fat droplets) in the liver increase with progression of fatty liver. Previous techniques have been unable to obtain information on multiple types of scatterers under observation at once, but these recent studies may enable high-speed evaluation of the dynamics and properties of various tissues. However, improvements in signal processing technology and multifaceted verification are required to realize this capability.

## Backscatter coefficient estimation

### Overview

The backscatter coefficient (BSC) is defined as the time-averaged scattered intensity in the backward direction per unit solid angle per unit volume, normalized by the time-averaged incident intensity. General B-mode generation and amplitude envelope statistics mainly use time information. However, backscattered signals are converted into the frequency domain for analysis in BSC estimation. BSC is frequency dependent, because ultrasonic scattering is affected by the intrinsic acoustic impedance and size of the scattering medium. In other words, BSC evaluation is a key method for estimating microstructural characteristics such as the shape, size, composition, and concentration of the tissue to be observed, as well as the impedance ratio between the scatterer and the surrounding medium.

### Evaluation of tissue structure

Sigelmann and Reid first developed a method for estimating backscatter power from a volume of randomly distributed scatterers using a single-element planar transducer [90]. Subsequently, D’Astous determined BSC by plane wave evaluation [91], and Insana and Hall further improved the accuracy of estimation [92, 93]. Insana proposed a BSC estimation method employing array transducers [94]. BSC is generally estimated as a parameter related to attenuation, as proposed by Yao [95] and by Huisman and Thijssen [96]. It is also possible to evaluate the effective scatterer diameter (ESD) and effective acoustic concentration (EAC) by parameterizing the BSC as a function of frequency [92, 93, 96–98].

### Clinical applications

BSC-based QUS has been applied to characterizing the tissue microstructure of the liver, prostate, pancreas, spleen, eye, and lymph nodes, among others [78, 79, 99–103]. Good results have been obtained by the diagnostic equipment (or specially developed scanners) available in each era from the 1980s to the present. However, the BSC evaluation method has continued to evolve as the frequency band of ultrasound used clinically has become extremely wide (on the high-frequency side), and as the acquisition accuracy of RF signals has improved due to digitalization. For example, Lavarello mentioned the limitations of traditional methods and proposed a new theory [104], and Franceschini and Cloutier proposed the effective medium theory combined with the polydisperse structure factor model to incorporate the polydispersity of aggregate size [105, 106].

Franceschini also proposed a method for calculating the BSC under arbitrary conditions in which the actual structure of living tissue is prepared and the acoustic impedance of each tissue is presented as a two-dimensional or three-dimensional computer model [105, 107]. This technology makes it possible to understand the frequency characteristics of the BSC in living tissues that have complex structures and were previously difficult to verify. Furthermore, because the BSC evaluation method including various attenuation corrections proposed so far and the verification of the evaluation accuracy of ESD and EAC will be realized, it is expected that the BSC evaluation method will be implemented in clinical equipment in the future.

**Fig. 3:**
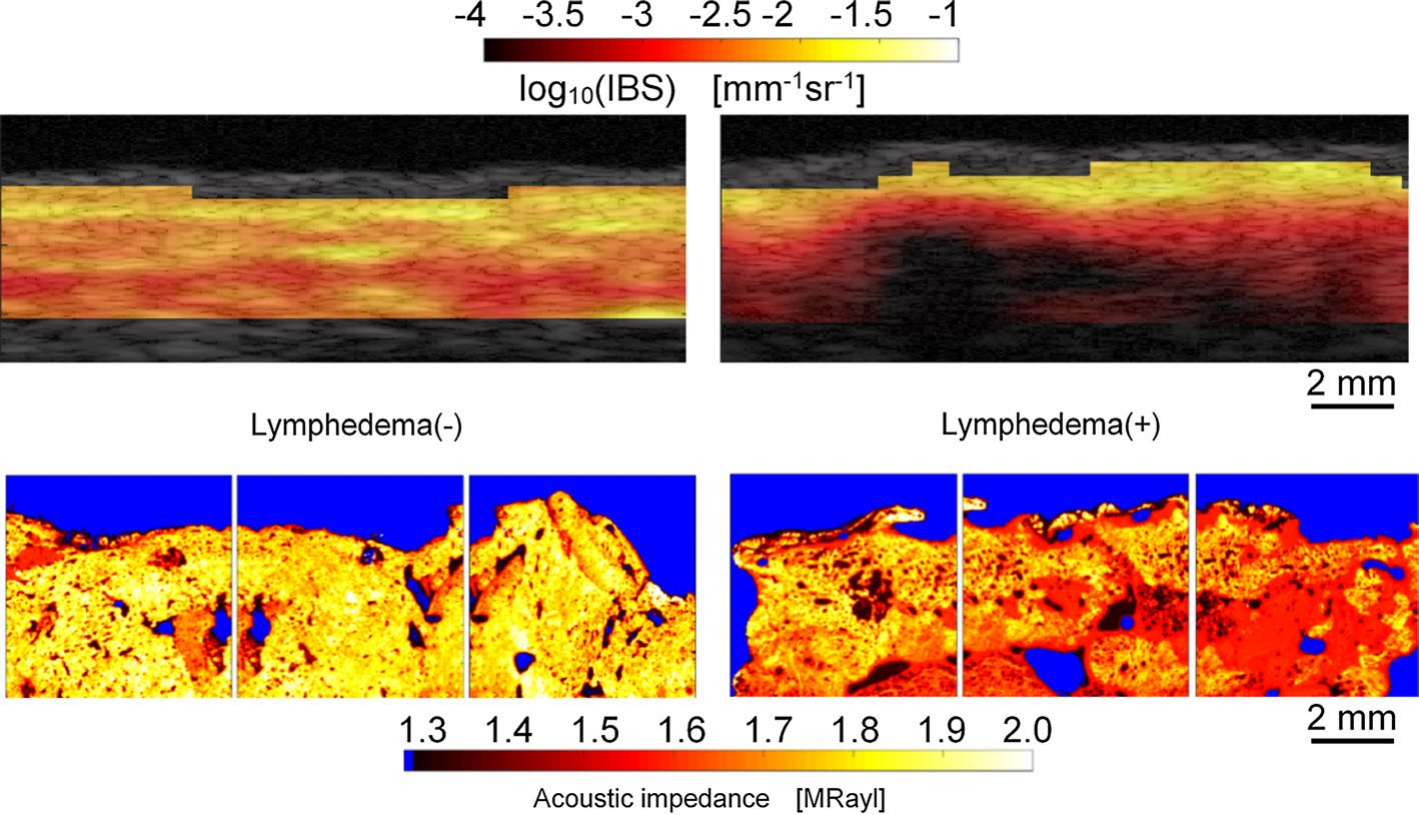
Parametric images (top) of integrated backscatter and acoustic impedance maps (bottom) of human skin dermis with (+) and without (−) lymphedema

Figure 3 shows an example of evaluation of human skin dermis with and without lymphedema [108], which confirms that BSC values are high in regions of high acoustic impedance. Verification of the relationship between acoustic impedance and BSC by Franceschini’s two-dimensional impedance map method confirms that the microacoustic characteristics at the microscopic level depend on the characteristics of the echo signal acquired by the diagnostic equipment.

## Challenges and prospects

### Ultrasonic biomarkers

Several QUS methods (including shear wave elastography and transient elastography) that use the acoustic properties of living tissues as evaluation parameters have been proposed and realized, as described in the present review. However, the relationships between the types of physical properties (or pathological level structure) of the biological tissue responsible for the image features and QUS parameters have not been sufficiently verified because of the variety of conditions under which living tissue is observed with ultrasound. In addition, discrepancies always exist between theoretical and actual measurements. A major problem in the clinical application of QUS is the dependency of the evaluation result on the acquisition conditions of the RF echo signal, which is the source of the image information and varies among diagnostic equipment. Dependency on the acquisition conditions and equipment has been a longstanding concern in medical imaging modalities such as CT and MRI, as well as in ultrasound. However, QUS studies have most commonly been conducted by independent researchers, and a comprehensive study has not been completed.

The Quantitative Imaging Biomarkers Alliance (QIBA) [109] was established in 2007 under the leadership of the Radiological Society of North America (RSNA) to overcome the problem of medical imaging modalities being unable to progress beyond the stage of subjective evaluation. QIBA is a network of health care workers such as medical doctors, as well as engineering researchers and equipment development manufacturers, that carries out major activities aimed at the establishment of medical imaging biomarkers. Three committees have been established in collaboration with the AIUM: the Contrast-Enhanced Ultrasound (CEUS), Ultrasound Shear Wave Speed (SWS), and Ultrasound Volume Blood Flow biomarker committees. In Japan, J-QIBA was established in 2015 as an initiative of the Japan Radiological Society (JRS) [110]. The Japanese Society of Ultrasonics in Medicine (JSUM) collaborates mainly on the standardization of shear wave elastography and also carries out its own activities [111–113]. QIBA and J-QIBA activities to standardize QUS parameters and establish ultrasonic biomarkers include evaluation of clinical data collected at various clinical facilities; in addition, these entities are active in the construction of standardized phantoms, establishment of computer simulation methods, and standardization of diagnostic protocols. In 2020, the Pulse-Echo Quantitative Ultrasound (PEQUS) Biomarker Committee [114] was established within QIBA in response to the successive implementation of attenuation and speed of sound evaluation technologies in ultrasonic diagnostic equipment. The PEQUS committee undertakes evaluation of attenuation and speed of sound methods that have already been implemented in clinical equipment, as well as examination of BSC and RF data collection methods.

### Micro-specific acoustic characteristics

To verify the accuracy of QUS methods for evaluating the properties of echo signals, it is necessary to understand the intrinsic acoustic characteristics of individual living tissues. Ultrasonic observation with a higher resolution than is possible at the clinical level is indispensable for this purpose, and is realized by scanning acoustic microscopy (SAM). SAM uses ultrasound frequencies of 100 MHz or higher. Spatial resolutions of 15 and 1.5 µm are obtained at frequencies of 100 MHz and 1 GHz, respectively, and it is possible to observe organelles at frequencies above 200–300 MHz.

**Fig. 4:**
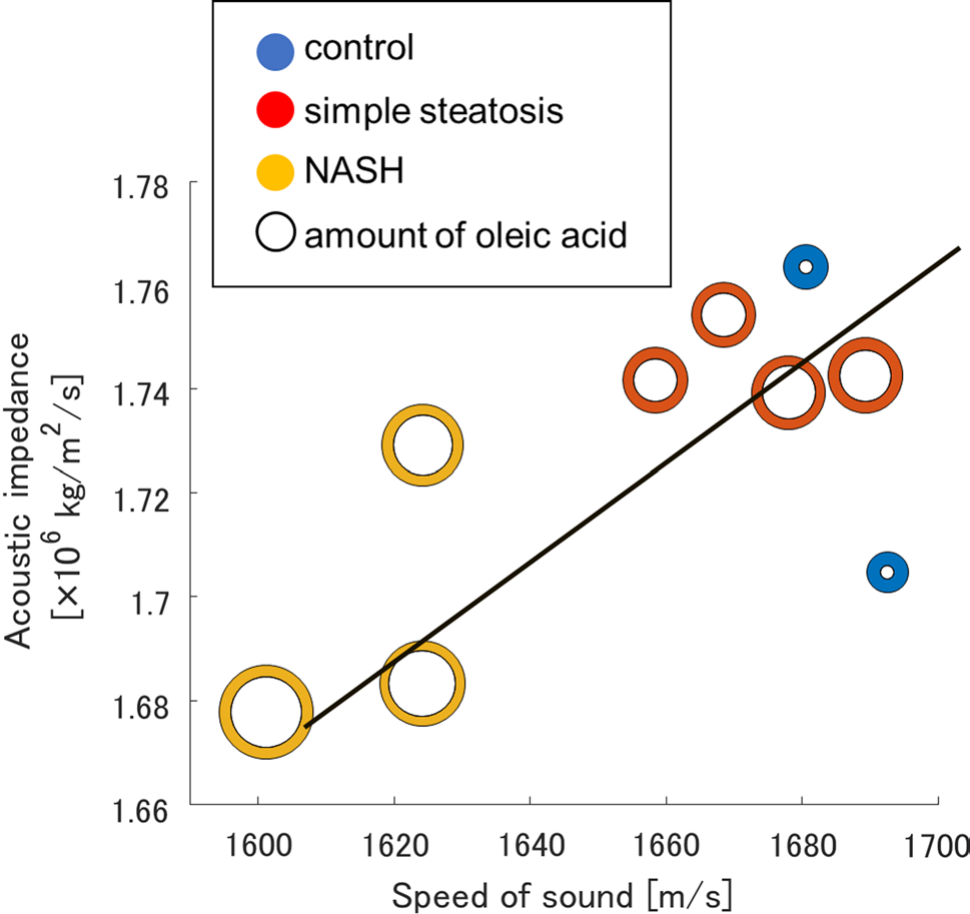
Relationship of speed of sound, acoustic impedance, and total amount of fatty acid content in control (blue), simple steatosis (red), and NASH (yellow) livers. White circles indicate the amount of oleic acid in the total amount of fatty acids

Liquid media or unstained tissue specimens sliced to 4–20 µm are commonly used to assess attenuation and speed of sound [115–118]. SAM can also be used to directly evaluate the acoustic impedance of extracted raw biological samples including living tissues such as cultured cells or acids, which is one of the main acoustic characteristics that determine the degree of attenuation and backscattering [119–122]. Figure 4 shows the relationship between the speed of sound, acoustic impedance, and amount of fatty acid content in control, simple steatosis, and nonalcoholic fatty liver disease (NASH) livers [119]. The figure confirms that compared with other liver types, NASH liver has a slower speed of sound and lower acoustic impedance, and an extremely high ratio of oleic acid to the total amount of fatty acids. This characteristic may enable the evaluation of canceration tendency using ultrasound.

**Fig. 5:**
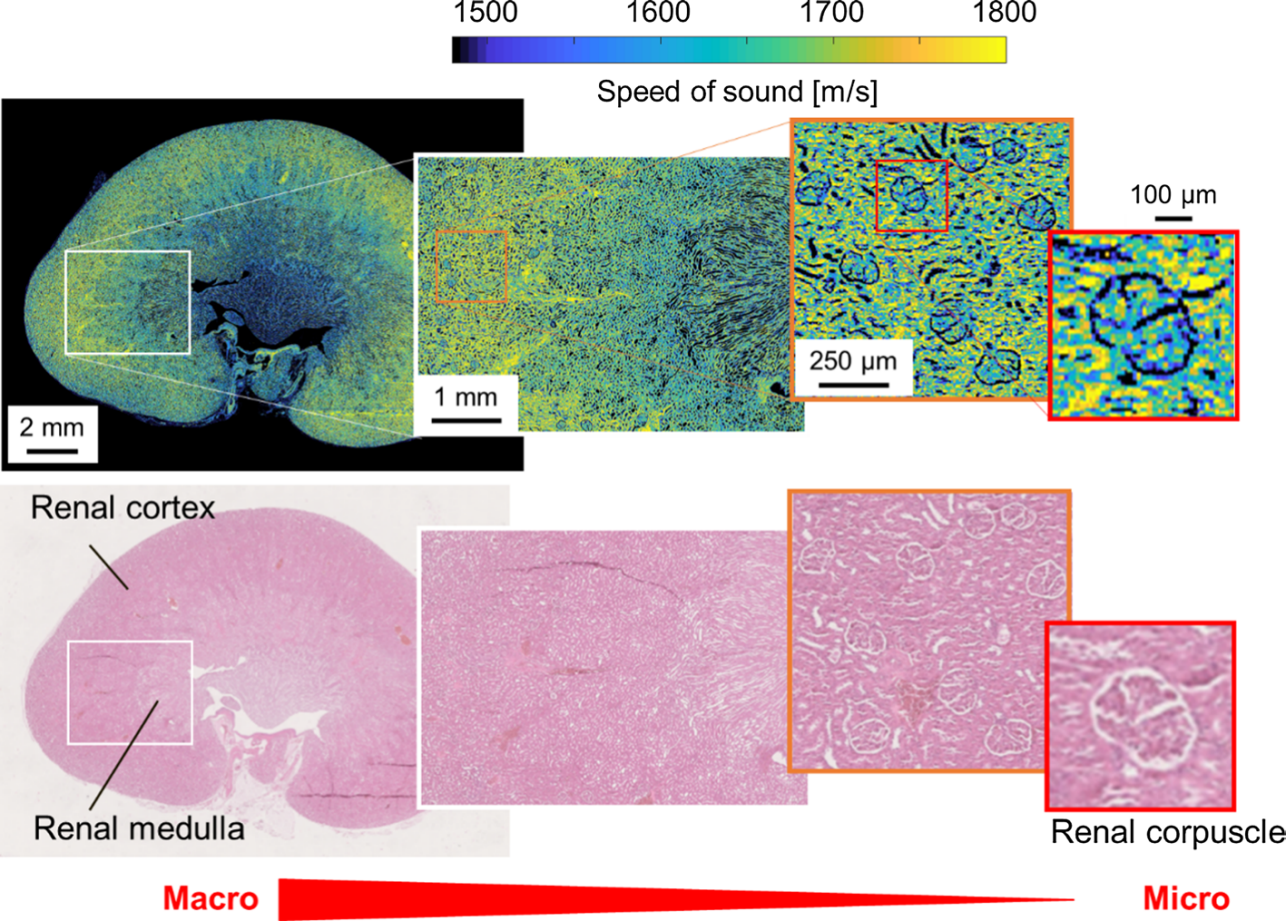
Multi-scale evaluation of speed of sound in rat kidney with 250-MHz ultrasound (top) and pathological images of the corresponding site (bottom)

In recent years, in addition to attenuation, speed of sound, and acoustic impedance, multiple indicators such as thickness, density, and bulk modulus have also been compared [123, 124]. Acoustic characteristics have also been evaluated in wide space and in wide frequency bands that correspond with those of in vivo QUS [108, 125]. As an example of the results of this technology, Fig. 5 shows the multi-scale evaluation of the speed of sound in rat kidney. Figure 5 shows the evaluation results only at 250 MHz. Even based on the results of evaluation using only a single frequency, the same tissue structure can be confirmed as in the pathological image, and physical differences in microtissues that cannot be detected in the pathological image can be understood. It is also possible to understand the multidimensional features by combining the evaluation results obtained at lower or higher frequencies [125]. These studies provide clues regarding the relationship between microscale acoustic properties and clinically observed structural-level living tissues. These are useful for providing direct physical quantities in the construction of three-dimensional impedance maps in BSC evaluation, and for multi-scale accuracy verification in evaluation of attenuation and amplitude envelope characteristics. In addition, the application of photo acoustics to this technology [126, 127] is expected to greatly contribute to the future development of QUS.

## Conclusion

Various QUS methods, particularly shear wave elastography, attenuation evaluation, and speed of sound evaluation have been developed and implemented in clinical equipment and are now being applied in a wide range of fields. The theory behind and full meaning of the QUS parameters produced by such equipment are not fully understood at present; however, large-scale projects (e.g., QIBA, J-QIB) are under way to address this problem, and working group activities are being actively promoted in the participating academic societies. Basic research to support theory and practice is also continuing to develop. It is important that users of the QUS method not only use the technology but also recognize that their research results will be added to large-scale standardization studies that are currently under way. In addition, considering the wide diversity of QUS methods, it is essential to conduct a thorough investigation into the suitability (or unsuitability) of each QUS method for the particular area to be evaluated. Despite current challenges, because QUS technology incorporates the properties and advantages of ultrasound, it undoubtedly has great potential for future application.
